# Cilostazol protects against degenerative cervical myelopathy injury and cell pyroptosis via TXNIP-NLRP3 pathway

**DOI:** 10.1186/s13008-024-00108-y

**Published:** 2024-01-17

**Authors:** Fei Xu, Zhuo Tian, Zhengguang Wang

**Affiliations:** 1 Department of Neck-Shoulder and Lumbocrural Pain, Yantai hospital of traditional Chinese medicine, 39 Xingfu Road, Zhifu District, Yantai, 264000 Shandong P.R. China; 2 Department of General Surgery, Yantai hospital of traditional Chinese medicine, Yantai, Shandong China

**Keywords:** Degenerative cervical myelopathy, Cilostazol, Pyroptosis, TXNIP, NLRP3

## Abstract

Degenerative cervical myelopathy (DCM) is one of the most common and serious neurological diseases. Cilostazol has protective effects of anterior horn motor neurons and prevented the cell apoptosis. However, there was no literatures of Cilostazol on DCM. In this study, we established the DCM rat model to detect the effects of Cilostazol. Meanwhile, the neurobehavioral assessments, histopathology changes, inflammatory cytokines, Thioredoxin-interacting protein (TXNIP), NOD‑like receptor pyrin domain containing 3 (NLRP3) and pro-caspase-1 expressions were detected by Basso, Beattie, and Bresnahan score assessment, Hematoxylin and Eosin Staining, Enzyme-linked immunosorbent assay, immunofluorescence and Western blotting, respectively. After treated with Cilostazol, the Basso, Beattie, and Bresnahan (BBB) score, inclined plane test and forelimb grip strength in DCM rats were significantly increased meanwhile the histopathology injury and inflammatory cytokines were decreased. Additionally, TXNIP, NLRP3 and pro-caspase-1 expressions levels were decreased in Cilostazol treated DCM rats. Interestingly, the using of siTXNIP significantly changed inflammatory cytokines, TXNIP, NLRP3 and pro-caspase-1 expressions, however there was no significance between siTXNIP and Cilostazol + siTXNIP group. These observations showed that Cilostazol rescues DCM injury and ameliorates neuronal destruction mediated by TXNIP/NLRP3/caspase-1 and pro-inflammatory cytokines. As a result of our study, these findings provide further evidence that Cilostazol may represent promising therapeutic candidates for DCM.

## Introduction

Degenerative cervical myelopathy (DCM), formerly known as cervical spondylotic myelopathy, is one of the most common and serious neurological disease caused by spinal cord compression induced degenerative vertebral column abnormalities with various symptoms including spinal cord injury, numbness of extremities and gait disturbance [1, 2]. Patients with DCM suffered with neck and shoulder pain, limited range of movement and upper motor neuron damage [3, 4]. With the development progress of modern clinical technology, the therapy for DCM including conservative treatment and surgically decompressing. Studies have shown that decompression surgery could prevent the development of DCM but with poor prognosis [5]. Moreover, studies have shown that spinal cord microvasculature disruption, inflammation and activation of apoptotic signaling pathways are involved in secondary spinal cord injury in DCM [6, 7]. Therefore, it is vital to investigate the potential mechanism and useful treatment of DCM.

As shown in various studies, the central nervous system caused inflammation was lasted in the progression of DCM [8]. Pyroptosis is a recently discovered mode of cell death in multiple tissues caused by inflammation, whether acute or chronic [9–11]. Pro-inflammatory programmed cell death is often mediated by inflammasomes, which release inflammatory mediators such as IL-1β and IL-18 [10, 12]. As a result, excessive inflammatory responses are triggered. As a result of activation of the NOD‑like receptor pyrin domain containing 3 (NLRP3) inflammasome, procaspase-1 is converted into cleaved caspase-1 meanwhile pro-IL-1β is converted into IL-1β mechanically [13]. It has been discovered that NLRP3-related pyroptosis plays a key role in DCM diseases, particularly in those involving tissue damage and inflammation [14, 15]. Furthermore, oxidative stress induces the activation of inflammasomes. In Central nervous system (CNS) pathologies, including post-ischemic pain, the NLRP3 signaling cascade is thought to play an important role in regulating neuroinflammatory responses.

It has been demonstrated that in addition to swelling, membrane lysis, chromatin fragmentation, and intracellular pro-inflammatory factors, pyroptosis is implicated in cell swelling. Multiple neurological disorders and spinal cord injuries are associated with pyroptosis-regulated cell death, according to current research [16]. Thioredoxin-interacting protein (TXNIP), plays an important role in activating NLRP3 inflammasomes, regulates oxidative stress, cell proliferation, differentiation, and apoptosis [17]. TXNIP/NLRP3 inflammasome play a key role in the pathogenesis of inflammation diseases and maintains redox balance in cells. Previous studies have shown that TXNIP exacerbates oxidative stress as well as increases inflammation in an NLRP3-dependent manner [18, 19].

Cilostazol, a potent inhibitor of phosphodiesterase III (PDE3), has been worldwide used for the treatment of lower extremity peripheral arterial disease and recurrent ischemic stroke [20, 21]. In addition, Cilostazol has shown to have protective properties in ischemia-reperfusion injury, hepatic steatosis and even neuroprotective properties in chronic compression of the cervical spinal cord and neuronal degeneration [22–24]. It has been reported that neuroprotective effects of Cilostazol were mediated by the anti-inflammatory, anti-oxidant and anti-apoptotic functions. Recent studies have revealed that Cilostazol could ameliorated spinal cord ischemia-reperfusion injury in rabbits [25]. Additionally, Cilostazol has protective effects of anterior horn motor neurons and prevented the cell apoptosis [24, 26]. Taken together, Cilostazol was neuroprotective in the DCM and was potentially useful in the treatment of DCM.

Currently, the neuroprotective effects of Cilostazol have been demonstrated in several disease conditions ravaging the central nervous system [27, 28]. In murine cerebral cortex following transient ischemia, Cilostazol ameliorates NLRP3-induced allodynia and hyperalgesia. The central post-ischemic pain problem is caused by the NLRP3, ASC, caspase-1, and IL-1β inflammasome, Cilostazol functions to inhibit these inflammasomes [29].

Based on the aforementioned literature, in the present study, we aimed to explored the effects of Cilostazol on DCM rat model. Furthermore, we investigated the effects of Cilostazol on inflammatory cytokines and TXNIP/NLRP3 relative proteins. This study aimed to provide a scientific reference for the research of the mechanism of Cilostazol and identify a novel therapeutic target for DCM.

## Results

### Cilostazol promoted neurobehavioral behavior recovery in DCM rats

**Fig. 1:**
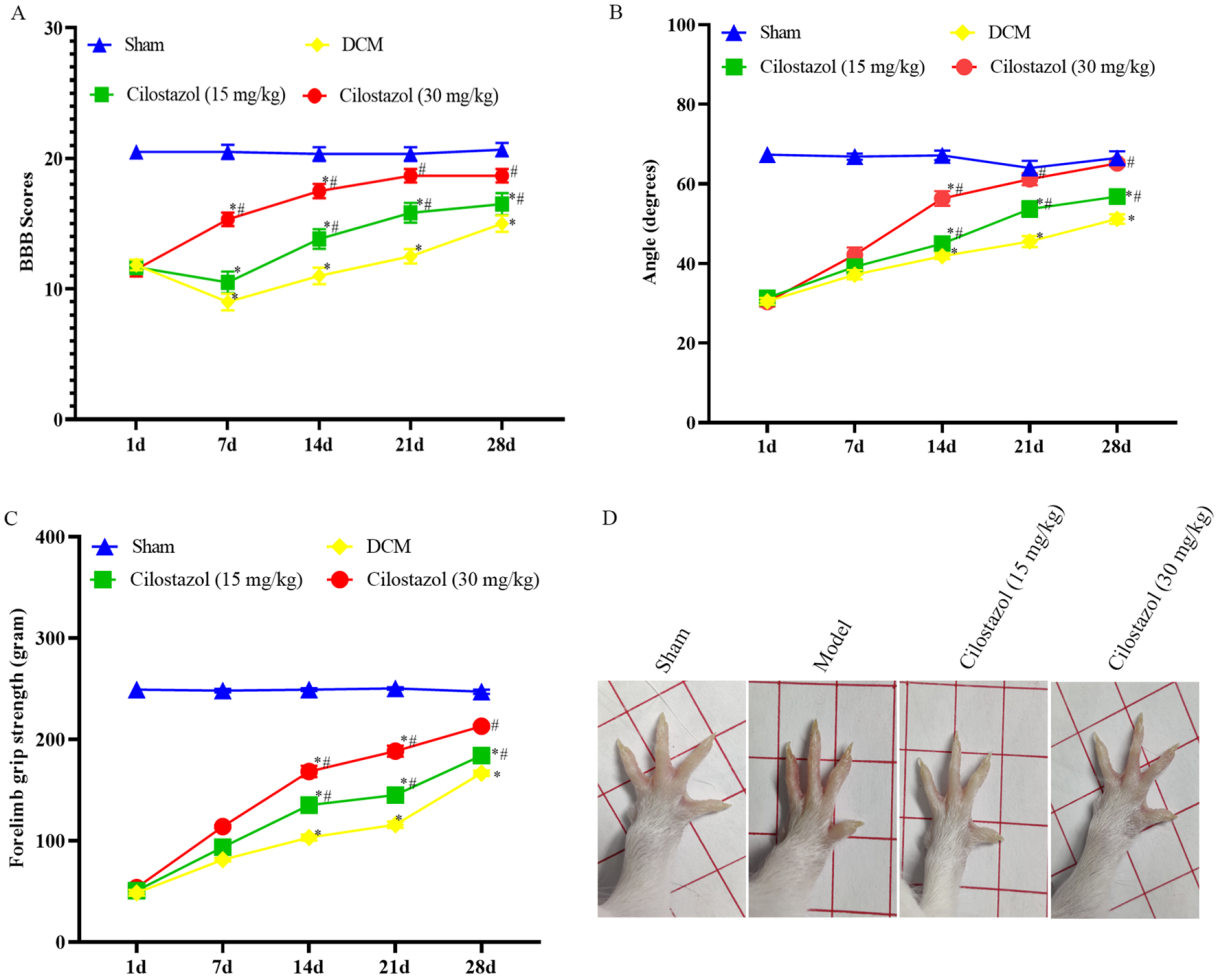
Cilostazol improves the motor function recovery in DCM model rats. **A** BBB scores; **B** Angle degree; **C**, **D** Forelimb grip strength; ^*^p < 0.05 vs. Sham group. ^#^*p* < 0.05 vs. DCM group

The cervical spinal cord of DCM rats was compressed after surgery. Cervical spinal cord compression caused hindlimb paralysis in DCM group, the locomotion of the 15 mg/kg and 30 mg/kg Cilostazol group gradually improved compared to the DCM group. According to Fig. 1, BBB score, inclined plane test, and forelimb grip strength deteriorated in DCM group, however the scores were higher in 15 mg/kg and 30 mg/kg Cilostazol groups (*p* < 0.05).

### Cilostazol reversed histopathology injury and inflammatory cytokines in DCM rats

**Fig. 2:**
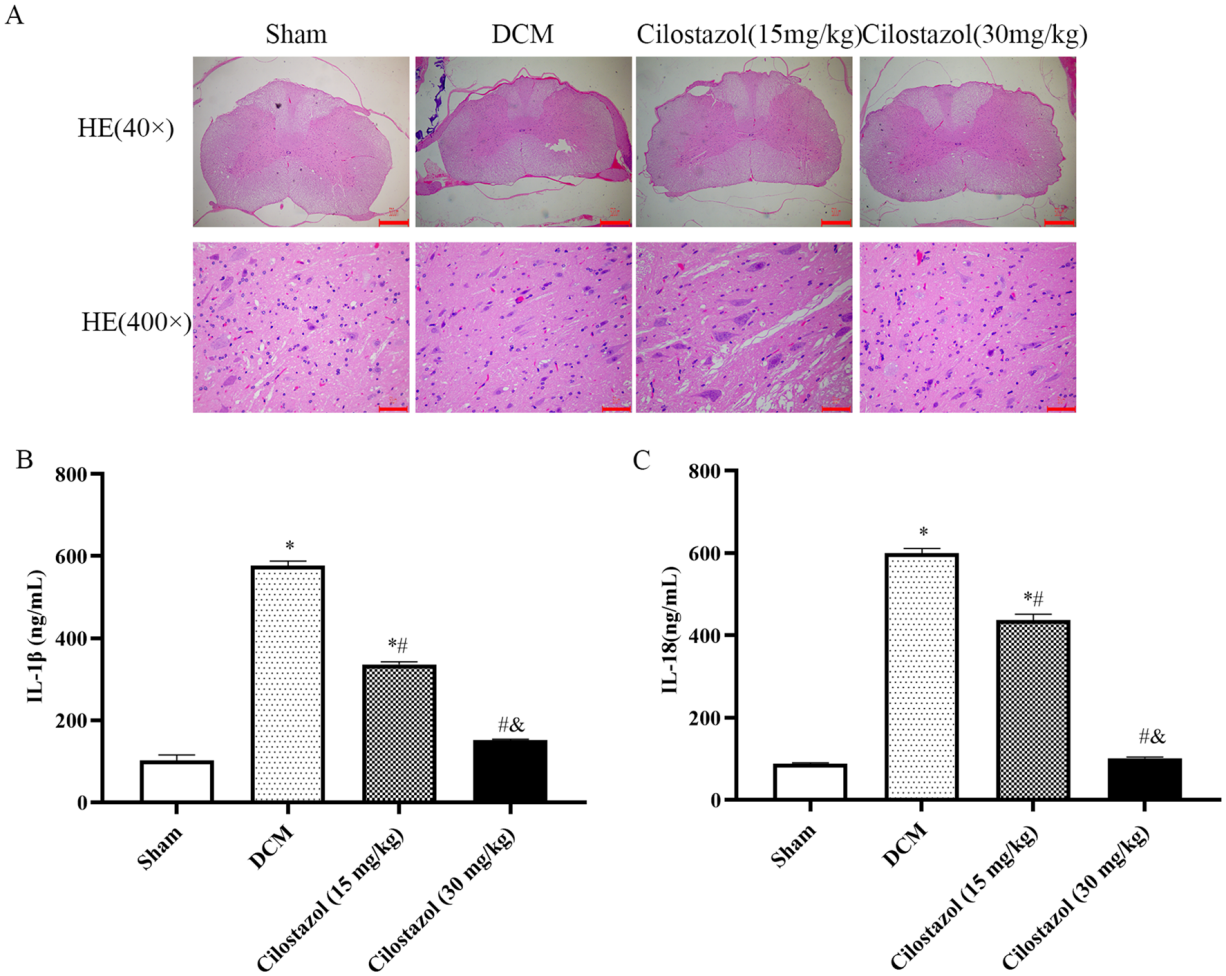
Spinal cord histopathology and level of inflammatory cytokines in DCM model rats. **A** Hematoxylin and eosin staining (Scar bar: 40×: 500 μm; 400×: 50 μm); **B** IL-1β; C: IL-18. ^*^p < 0.05 vs. Sham group; ^#^*p* < 0.05 vs. DCM group; ^&^*p* < 0.05 vs. Cilostazol (15 mg/kg) group

According to Fig. 2, HE staining revealed structural and neuronal damage, pyknosis of neuronal nuclei, and interstitial edema in the DCM group rat. Interestingly, Cilostazol reversed the tissue damage and inflammatory signs caused by DCM at doses of 15 and 30 mg/kg.

Contrast with the histopathology results, as shown in Fig. 2, according to the ELISA results, compared with the Sham group, inflammatory cytokines (IL-1β and IL-18) levels were significantly higher in the DCM group (576.82 ± 11.22 ng/mL, 599.53 ± 12.02 ng/mL). Meanwhile, the levels of IL-1β and IL-18 were significantly lower in the 15 and 30 mg/kg Cilostazol groups compared with the DCM group (*p* < 0.05). Meanwhile, 30 mg/kg Cilostazol showed lower levels of IL-1β and IL-18 than 15 mg/kg Cilostazol (*p* < 0.05).

### Cilostazol attuned GAP-43 and Iba-1 expressions in DCM rats

Growth-associated protein (GAP-43), a growth-related factor, is thought to play a role in differentiation and maturation of nerve cells. In the CNS, GAP-43 is highly expressed during axonal growth. According to the results of the present study, the DCM procedure reduced spinal levels of GAP-43 by a significant amount, confirming the impairments caused by DCM. It is interesting to note that compared to the DCM group (15.80 ± 1.42), Cilostazol administration significantly increased spinal levels of GAP-43 to a greater degree (15 mg/kg Cilostazol: 37.56 ± 2.83; 30 mg/kg Cilostazol: 41.54 ± 1.03), exhibiting the potential for improving the condition.

**Fig. 3:**
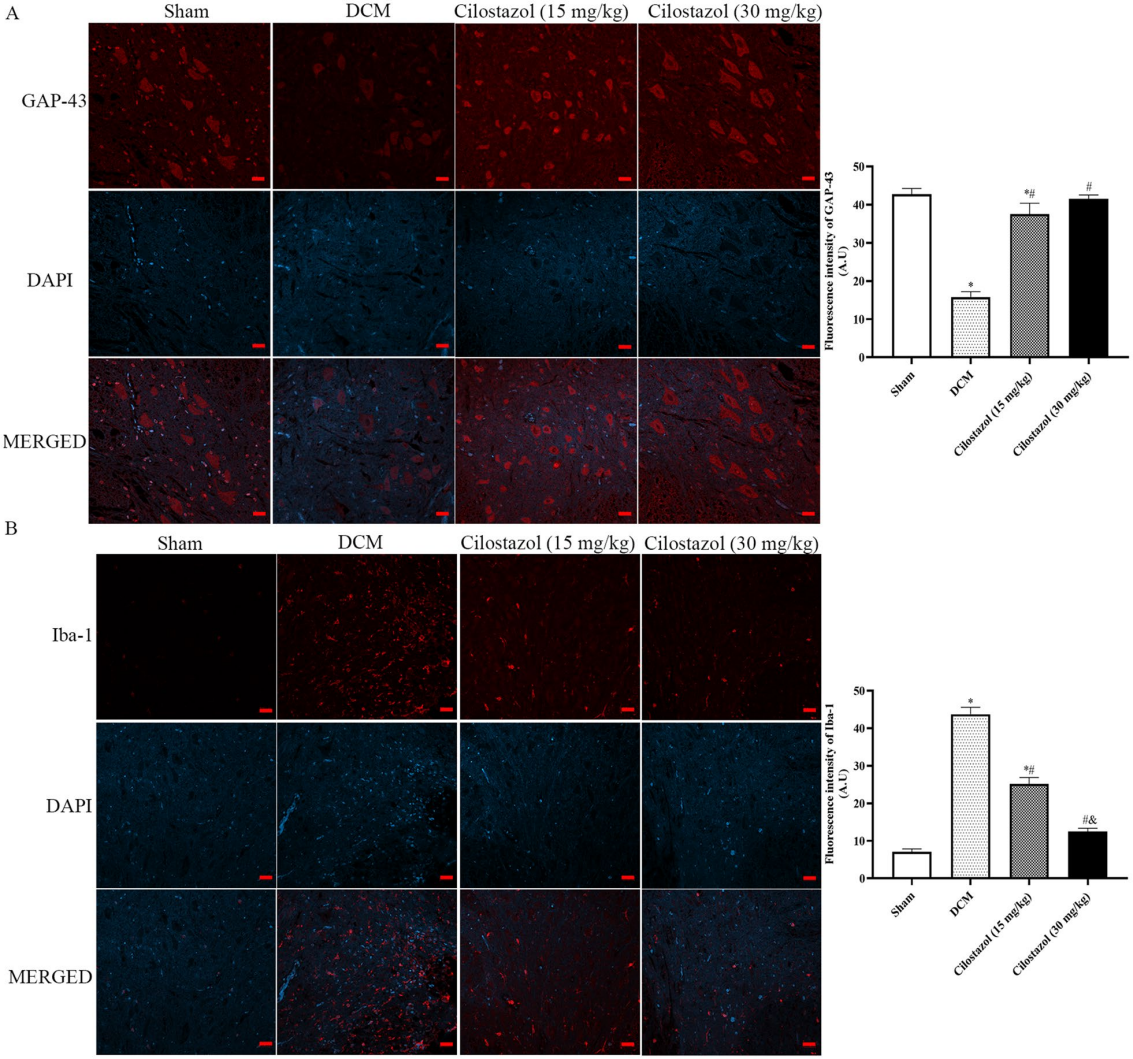
Effects of Cilostazol on GAP-43 and Iba-1 expressions in DCM rats were detected by immunohistochemistry (Scar bar: 20 μm). **A** GAP-43 expressions; **B** Iba-1 expressions. ^*^p < 0.05 vs. Sham group; ^#^*p* < 0.05 vs. DCM group; ^&^*p* < 0.05 vs. Cilostazol (15 mg/kg) group

Iba-1 antibody (microglia marker) was used to examine the effects of Cilostazol on inflammatory responses in spinal cord tissue. In the DCM group, Within the injury epicenter and adjacent areas, Iba-1 levels increased significantly (43.72 ± 1.87). According to Fig. 3, Cilostazol treatment significantly reduced microglial activation. According to the data, Cilostazol may reduce the initial activation of Iba-1 positive immune cells, thereby contributing to tissue repair. Meanwhile, 30 mg/kg Cilostazol showed lower levels of Iba-1 than 15 mg/kg Cilostazol (*p* < 0.05).

### Cilostazol attuned TXNIP, NLRP3 and cleaved caspase-1 expressions in DCM rats

In order to detect TXNIP, NLRP3 and cleaved Caspase-1 expressions in DCM rats, immunofluorescence analysis and western blot assay were performed.

**Fig. 4:**
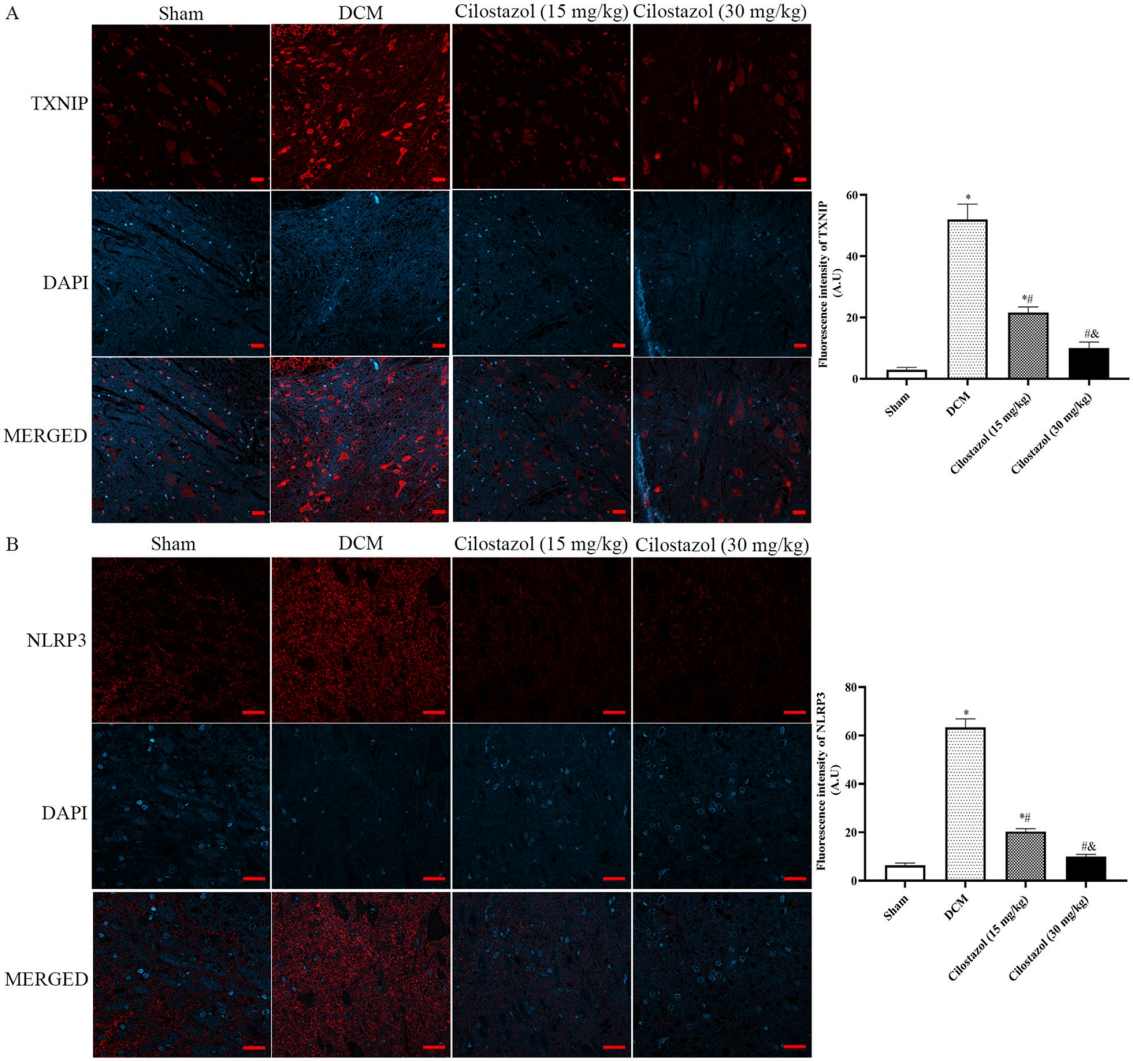
Effects of Cilostazol on TXNIP and NLRP3 expressions in DCM rats were detected by immunohistochemistry (Scar bar: 20 μm). **A** TXNIP expressions; **B** NLRP3 expressions. ^*^p < 0.05 vs. Sham group; ^#^*p* < 0.05 vs. DCM group; ^&^*p* < 0.05 vs. Cilostazol (15 mg/kg) group

**Fig. 5:**
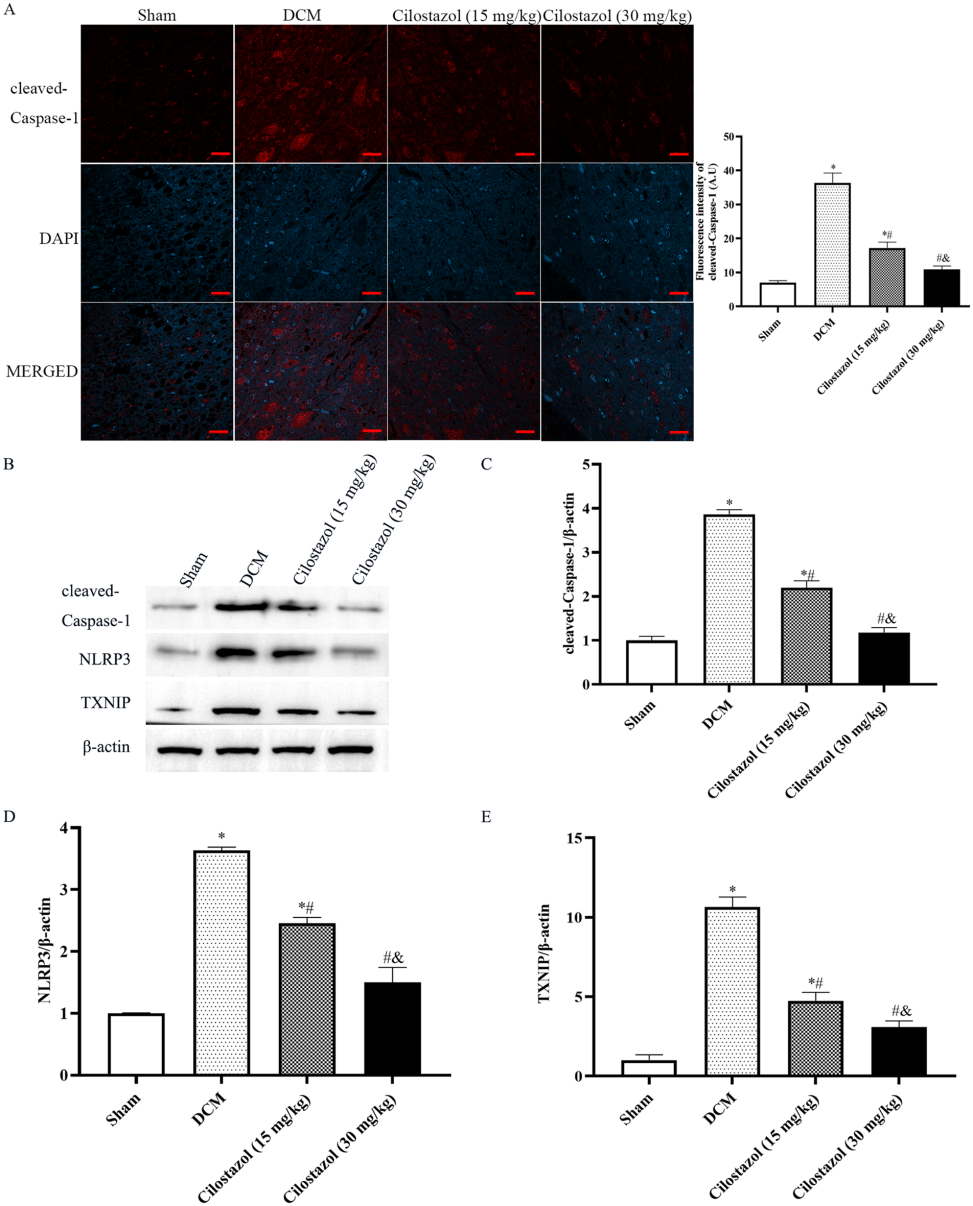
Effects of Cilostazol on cleaved-Caspase-1, TXNIP and NLRP3 expressions in DCM rats (Scar bar: 20 μm). **A** TXNIP expression was detected by immunohistochemistry; **B** Western blot images; **C** cleaved-Caspase-1 expressions; **D** TXNIP expressions; **E** NLRP3 expressions. ^*^p < 0.05 vs. Sham group; ^#^*p* < 0.05 vs. DCM group; ^&^*p* < 0.05 vs. Cilostazol (15 mg/kg) group

As shown in Figs. 4 and 5A, after DCM treatment, TXNIP, NLRP3, and cleaved Caspase-1 expressions were significantly increased.

Consistent with immunofluorescence study, as shown in Fig. 5, after DCM treatment, TXNIP, NLRP3, and cleaved Caspase-1 expressions were significantly increased. Compared with the DCM group, 15 mg/kg Cilostazol and 30 mg/kg Cilostazol could significantly decrease TXNIP, NLRP3, and cleaved Caspase-1 levels (*p* < 0.05). Meanwhile, 30 mg/kg Cilostazol showed lower levels of TXNIP, NLRP3, and cleaved Caspase-1 than 15 mg/kg Cilostazol (*p* < 0.05). Hence, we determined that Cilostazol decreases damage in DCM injury and shows a protective effect by reducing the TXNIP-NLRP3 interaction.

### Cilostazol attuned IL-1β/NeuN, cleaved-caspase-1/NeuN, NLRP3/NeuN and TXNIP/NeuN expressions in DCM rats

To confirm that Cilostazol could protect against DCM injury in a TXNIP-depenent way We silenced TXNIP by using siRNA. 30 mg/kg Cilostazol was used for the following experiments.

**Fig. 6:**
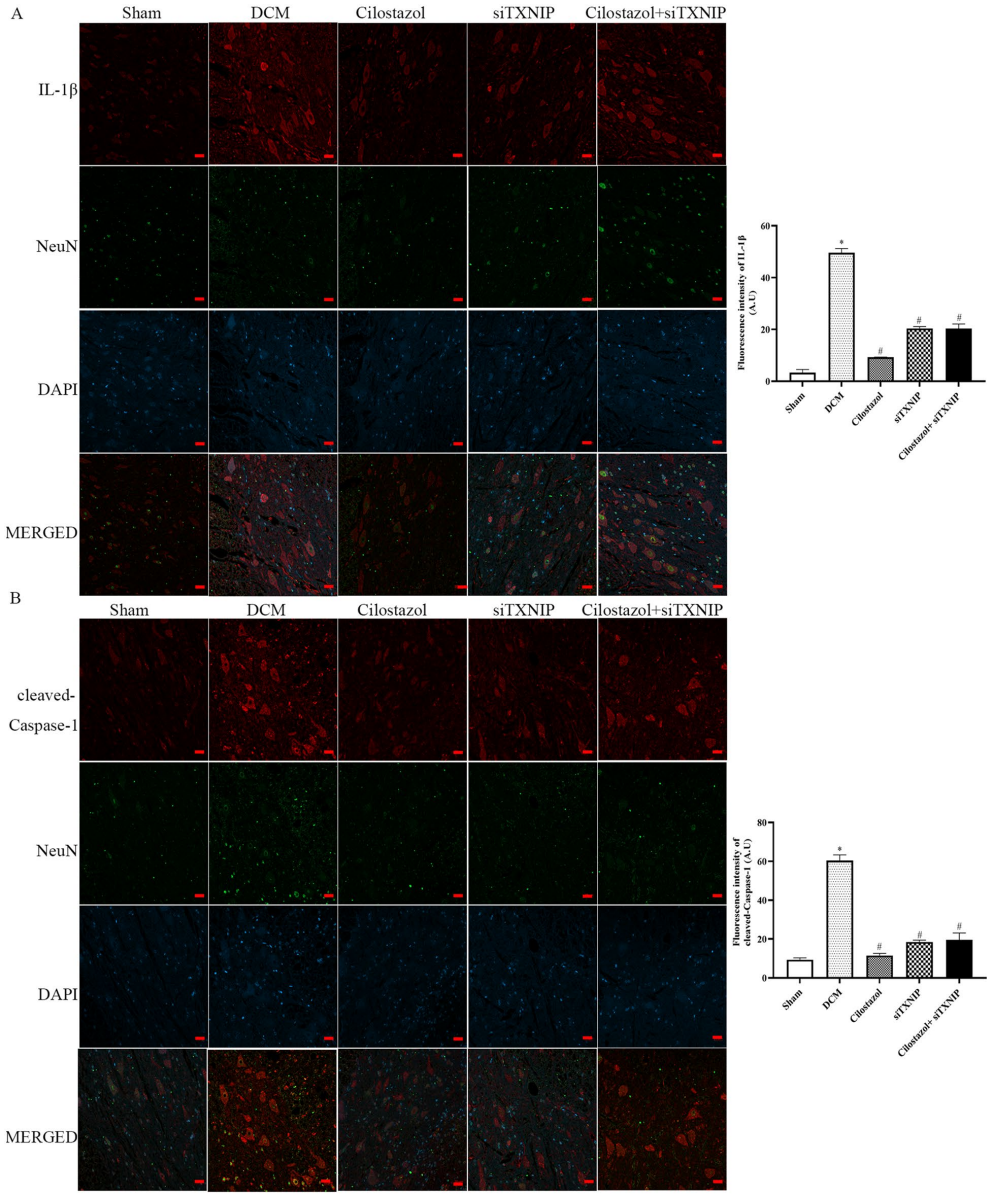
Effects of Cilostazol on IL-1β/NeuN and cleaved-Caspase-1/NeuN expressions in DCM rats were detected by immunohistochemistry (Scar bar: 20 μm). **A** IL-1β/NeuN expressions; **B** cleaved-Caspase-1/NeuN expressions. ^*^p < 0.05 vs. Sham group; ^#^*p* < 0.05 vs. DCM group; ^&^*p* < 0.05 vs. Cilostazol (15 mg/kg) group

**Fig. 7:**
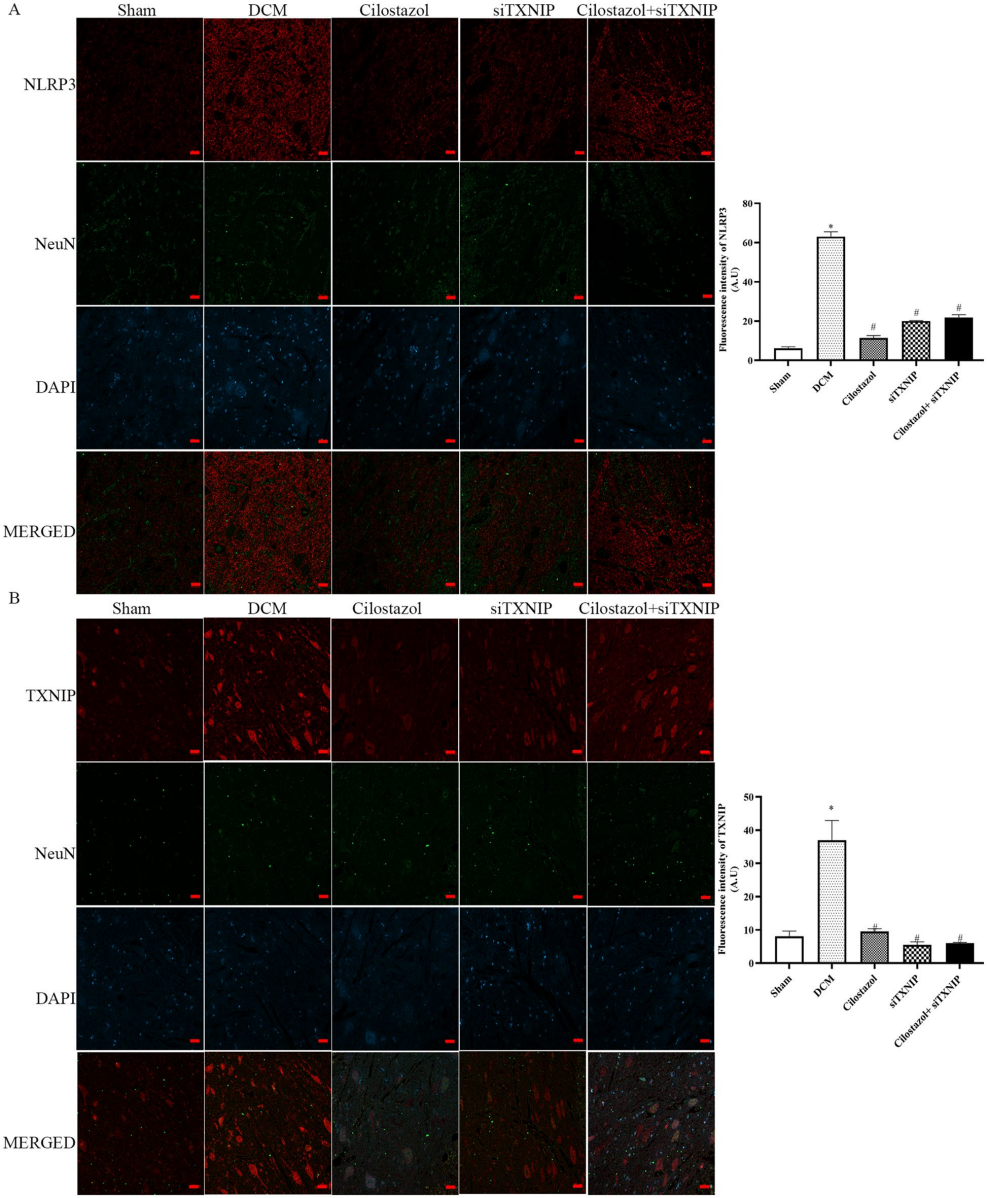
Effects of Cilostazol on NLRP3/NeuN and TXNIP/NeuN expressions in DCM rats were detected by immunohistochemistry (Scar bar: 20 μm). **A** NLRP3/NeuN expressions; **B** TXNIP/NeuN expressions. ^*^p < 0.05 vs. Sham group; ^#^*p* < 0.05 vs. DCM group

Immunofluorescence was used to determine the fluorescence intensities of IL-1β/NeuN, cleaved-caspase-1/NeuN, NLRP3/NeuN, and TXNIP/NeuN in the anterior horn of the lesioned area (Figs. 6 and 7). Additionally, Western blotting was used to analyze IL-1β, cleaved-Caspase-1 and NLRP3 in the lesioned spinal cords.

The immunofluorescence results showed that cleaved-Caspase-1, NLRP3, TXNIP and IL-1β were expressed in neurons. Meanwhile, the mean gray values of IL-1β, IL-18, cleaved-Caspase-1, NLRP3 and TXNIP were increased in the DCM group. Cilostazol administration significantly decreased spinal levels of IL-1β, cleaved-Caspase-1, NLRP3 and TXNIP to a greater degree, exhibiting the potential for improving the condition. It is interesting to note that when treated with siTXNIP, the levels of IL-1β, IL-18, cleaved-Caspase-1, NLRP3 and TXNIP were decreased. However, in the groups in which TXNIP was silenced, Cilostazol treatment (Cilostazol + siTXNIP group) showed no difference compared to siTXNIP group. The findings demonstrated that the protective effect of Cilostazol was reversed upon knockdown of TXNIP. Moreover, the protective effects of Cilostazol were observed to be dependent on the presence of TXNIP.

**Fig. 8:**
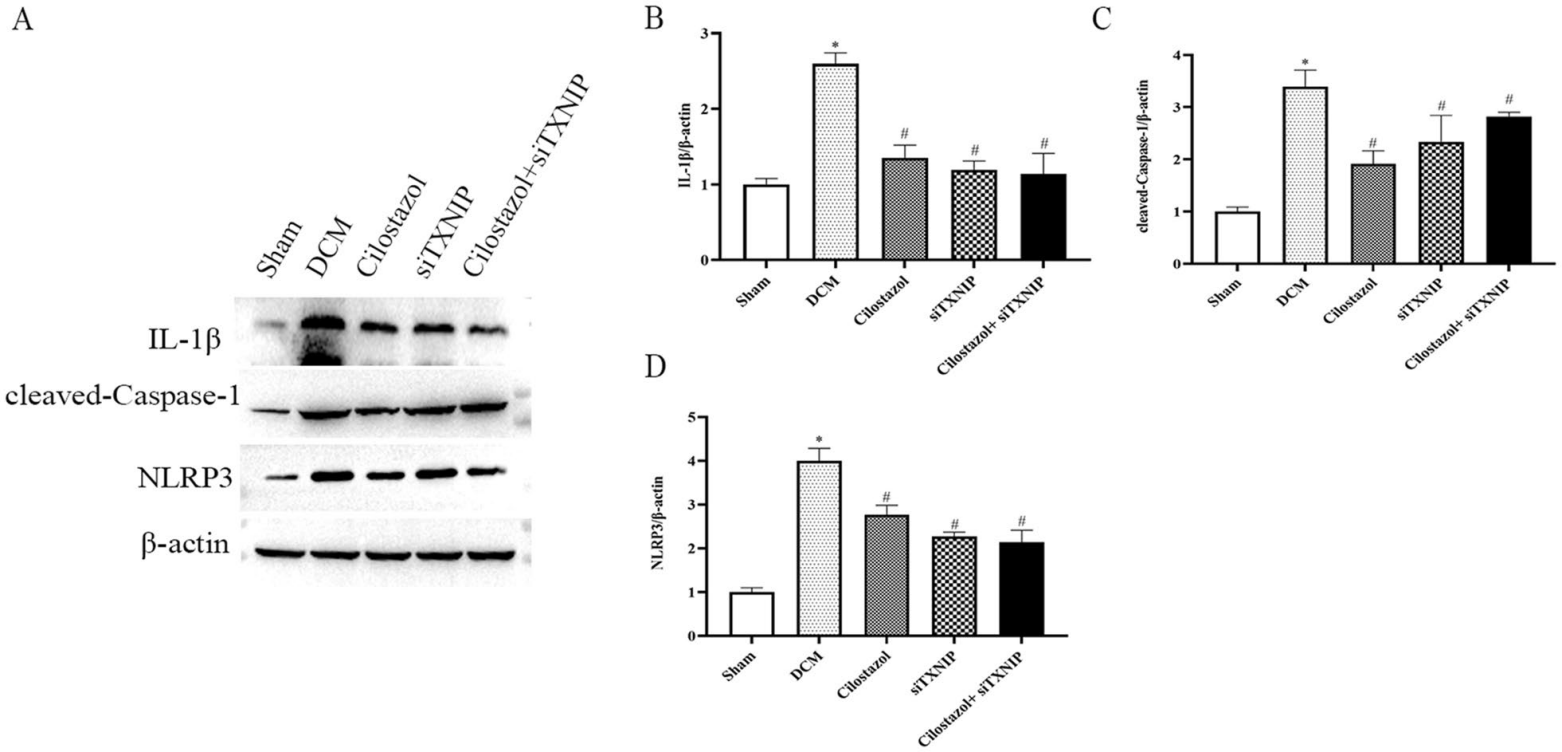
Effects of Cilostazol on IL-1β, IL-18, cleaved-Caspase-1 and NLRP3 expressions were detected by Western blot. **A** Western blot images; **B** IL-1β expressions; **C** cleaved-Caspase-1 expressions; **D** NLRP3 expressions. ^*^p < 0.05 vs. Sham group; ^#^*p* < 0.05 vs. DCM group

Using western blotting, we detected IL-1β, IL-18, cleaved-Caspase-1 and NLRP3 expressions. According to Fig. 8, compared with the DCM group, Cilostazol administration significantly decreased spinal levels of IL-1β (1.35 ± 0.17), cleaved-Caspase-1 (1.91 ± 0.25) and NLRP3 (2.77 ± 0.21), the western blot results were consistent with the immunohistochemistry.

In the groups in which TXNIP was silenced, Cilostazol treatment showed no difference compared to siTXNIP group. This observation indicates that knockdown of TXNIP counteracts the beneficial impact of Cilostazol. In conclusion, we confirmed that Cilostazol protects against DCM injury by reducing inflammation and pyroptosis in a TXNIP-depenent manner via downregulating the TXNIP-NLRP3 pathway.

## Discussion

DCM is a challenging spinal disease caused by compression and affects the normal life of patients [30]. To detect the effects of Cilostazol, we developed a DCM rat model. In DCM rats, Cilostazol promoted neurobehavioral behavior recovery while reversing histopathology injury and inflammation. Cilostazol significantly increased spinal levels of GAP-43 to a greater degree and reduce the initial activation of Iba-1 positive immune cells, exhibiting the potential for improving the condition. Meanwhile, Cilostazol could significantly decrease TXNIP, NLRP3, and cleaved Caspase-1 levels. To explore the mechanisms, a plasmid transfection of TXNIP was used. As shown in the results, Cilostazol could attuned the IL-1β, IL-18, cleaved-Caspase-1 and NLRP3 expressions through inhibiting the expressions of TXNIP.

Previous reports showed that pretreatment with Cilostazol significantly attenuated ischemia-reperfusion induced neuronal histopathologic injury meanwhile ameliorated the neurologic deficits by attenuating oxidative stress in rabbit and rat model [31, 32]. What’s more, administration of Cilostazol at a clinical dose could prevent the development of symptomatic myelopathy meanwhile attenuated neuronal loss and deterioration [33, 34]. All these results revealed the potential effects of Cilostazol on chronic cord compression. Consistent with the previous studies, our research revealed that Cilostazol could promote the neurobehavioral behavior recovery and attenuated histopathology injury in DCM rat model.

After DCM injury, the spinal cord got injured and the injury triggers a complex series of cell apoptosis and inflammation [35]. Many investigations showed that neuronal pyroptosis play key roles in the spinal cord injury induced inflammatory and recovery of the tissue integrity [36].

Pyroptosis was recently identified inflammatory cell death mediated by the inflammasome and dependent on caspase-1 [37]. In response to microbial infection and cellular damage, inflammasomes form in the cytosol. Inflammasome-mediated neuroinflammation in microglia is mediated by NLRP3, formed by five PRRs (NLRP1, NLRP3, NLRC4, Pyrin, and AIM2) [38, 39]. By assembling an inflammasome, procaspase-1 is proteolytically cleaved into active caspase, IL-1β and IL-18 are converted from their precursors into mature and biologically active products. There are many immune reactions that are mediated by mature IL-1β [40]. Pyroptosis, a form of proinflammatory cell death, is also induced by IL-1β or active caspases [41]. As soon as pyroptosis is activated, caspase-1 processes the precursor of the inflammatory cytokines IL-1β and IL-18 [42]. In this study, we found that after DCM, the spinal cord displayed characteristic features of pyroptosis, as evidenced by increased levels of cleaved- caspase-1, IL-1β, IL-18 and NLRP3. As a result of Cilostazol treatment, DCM-induced cell pyroptosis was decreased, suggesting that Cilostazol is a cellular mechanism that inhibits pyroptosis.

As an endogenous negative modulator of thioredoxin, TXNIP plays a crucial role in maintaining redox balance in cells [12, 43]. In addition to exacerbating oxidative stress, TXNIP also induces an inflammatory response in an NLRP3-dependent manner, which is also called the TXNIP-NLRP3 axis [17]. It was reported that Cilostazol significantly reduced NLRP3 inflammasome activation, as well as other harmful factors including TXNIP, IL-1β and IL-18 in human vascular endothelial cells [44]. In the current study, we found that TXNIP-NLRP3 axis was found to be involved in Cilostazol’s protective effect. Furthermore, we discovered that the TXNIP-NLRP3 axis caused DCM-induced pyroptosis, which could be the cause of excessive inflammatory injury. Interestingly, when we set the siTXNIP group, there was no significance between siTXNIP and Cilostazol + siTXNIP group. The results revealed that knockdown of TXNIP reversed the protective effect of Cilostazol.

## Conclusion

In this research, we investigated that under the condition of DCM, the markers of pyroptosis were activated and the expression of inflammation factors were increased. Meanwhile, the treatment with Cilostazol significantly decreased the marker levels of inflammasome meanwhile decreased the levels of TXNIP. Interestingly, the using of siTXNIP significantly changes levels of cleaved- caspase-1, IL-1β, IL-18 and NLRP3, however there was no significance between siTXNIP and Cilostazol + siTXNIP group. These observations showed that Cilostazol could alleviated the DCM injury via regulating TXNIP-NLRP3 pathway. Cilostazol may provide a novel treatment for DCM, thus providing a theoretical basis for future academic and clinical research.

## Materials and methods

### Chronic cervical cord compression model

The animal experiments were carried out in compliance with the ARRIVE guidelines (Animal Research: Reporting of In Vivo Experiments). We obtained approval of the Animal Ethics Committee of Yantai hospital of traditional Chinese medicine for the procedures we used in this study. The study was carried out according to the principles of Guide for the Care and Use of Laboratory Animals published by the National Institutes of Health (NIH Publications No. 8023). We used 90 Sprague-Dawley (SD) rats (weighing 250–300 *g*) with a 12-hour light/dark cycle standard condition. The sample size was determined according to the previous study [45].

Pentobarbital (40 mg/kg) was used to anesthetize the rats and induce chronic compression in the cervical cord. A posterior approach was used to expose the spinal process and laminae of C4–C6, followed by the ligamentum flavum and C5 lamina were removed to the epidural space. In order to implant the same polyvinyl alcohol-polyacrylamide hydrogel at the C6 level on the left side of the spinal canal, the rats underwent a careful procedure. After expansion of the hydrogel, the spinal cord gradually compressed (about 4 weeks). Sham group rats underwent sham surgery (only received ligamentum flavum and C5 lamina removed to the epidural space without sublaminar placement) [46].

 To evaluate the establishment of DCM model rats, the rats were assessed by Behaviors test and all the rats were selected according to the DCM priori inclusion/exclusion criteria [47]. Then, the DCM model rats were randomly divided into three groups (*n* = 10). In this study, there were four groups (*n* = 10): Sham group, DCM model group (DCM), 15 mg/kg Cilostazol group for DCM models treated with 15 mg/kg/day, 30 mg/kg Cilostazol group for DCM models treated with 30 mg/kg/day. Cilostazol was orally administered to the rats once a day. The drug was administered for 5 weeks. Investigators were blinded to group allocation during the experiment and analysis.

### Neurobehavioral assessments

#### Basso, beattie, and bresnahan (BBB) score

An assessment of motor function based on a 21-point scoring system was used to assess the locomotor recovery after DCM [48]. Animals on an open field were observed to move hindlimb joints, place paws during stepping, support weight, and coordinate forelimbs and hindlimbs independently by two examiners blinded to the experimental conditions.

### Inclined plane test

On an inclined plane ranging from 0° to 90°, rats are tested on their maximum slope of stability [49].

### Forelimb grip strength assessment

The grip strength of rats was assessed using forelimb grip strength assessment. In the present study, experimenters were blinded to the treatment conditions during all behavioral studies [49].

### Hematoxylin and eosin staining

Following behaviors evaluation, rats were euthanized with pentobarbital (40 mg/kg) intraperitoneally. A 4% paraformaldehyde solution was applied to the spinal cords for 12 h at 4 ℃. According to the previous study, the experimental procedure was performed. A magnification of 40× and 400× was used to observe the sections under microscopic (Olympus, Tokyo, Japan).

### Immunofluorescence analysis

In accordance with the manufacturer’s instructions, the expression of GAP-43, Iba-1, TXNIP, cleaved-Caspase-1 and NLRP3 expressions in four to six lumbar cord segments (L4-L6) were detected by immunofluorescence. Then, the samples were blocked with 1% BSA and 0.1% Triton X-100 for 60 min, GAP-43, Iba-1, TXNIP, cleaved-Caspase-1 and NLRP3 (1:500, Cell signaling, Beverly, MA, USA) antibodies were added. Secondary antibodies IgG (H + L) FITC (1:1000, 11–4011-85, ThermoFisher) were added and incubated in the dark for 2 h. The images were taken and quantified with the A Zeiss LSM 800 confocal laser scanning microscope at a magnification of 20×.

### Enzyme-linked immunosorbent assay (ELISA)

At the end of the experiment, cerebrospinal fluid (CSF) was collected from the rats. 

We measured the concentrations of interleukin (IL)-1β and IL-18 in rat serum using ELISA kits. The experiment was conducted according to the manufacturer’s instructions.

### Western blotting

Based on previous studies, western blot protocols were followed [50]. In brief, using RIPA Lysis Buffer, total protein lysates of spinal cord tissues were prepared. BCA protein concentration kit was used to measure the protein concentration. Electrophoresis on 10% SDS gel resolved equal amounts of protein, which was then transferred to nitrocellulose. After blocking in 5% non-fat milk, Membranes were probed overnight at 4 ℃ with specific anti- SOCS1, TXNIP, Cleaved-Caspase-1 and NLRP3 (1:500, Cell signaling, Beverly, MA, USA). Membranes were then incubated with horseradish peroxidase-conjugated secondary antibodies for 60 min. A chemiluminescence detection system was then used to detect antibody binding.

### Statistical analysis

IBM SPSS Statistics Version 19.0 (SPSS I nc., Chicago, IL, USA) was used for all experimental data analysis. At least three sets of data were collected from each experiment and are expressed as means ± standard deviation. One-way analysis of variance (ANOVA) and Tukey’s post-test were used to analyze the significant differences between the two sets of data. Statistical significance was determined by *p* < 0.05.

## Data Availability

No datasets were generated or analysed during the current study.
